# A Scoping Review of the Role of Metalloproteinases in the Pathogenesis of Autoimmune Pemphigus and Pemphigoid

**DOI:** 10.3390/biom11101506

**Published:** 2021-10-13

**Authors:** Nicola Cirillo, Stephen S. Prime

**Affiliations:** 1 Melbourne Dental School, University of Melbourne, 720 Swanson Street, Carlton, Melbourne, VIC 3053, Australia; 2 Centre for Immunology and Regenerative Medicine, Institute of Dentistry, Barts and the London School of Medicine and Dentistry, Queen Mary University of London, London E1 4NS, UK; stephensprime@gmail.com

**Keywords:** pemphigus vulgaris, bullous pemphigoid, metalloproteinases, extracellular matrix, cadherins, integrins, cell adhesion

## Abstract

Pemphigus and pemphigoid diseases are potentially life-threatening autoimmune blistering disorders that are characterized by intraepithelial and subepithelial blister formation, respectively. In both disease groups, skin and/or mucosal blistering develop as a result of a disruption of intercellular adhesion (pemphigus) and cell-extracellular matrix (ECM) adhesion (pemphigoid). Given that metalloproteinases can target cell adhesion molecules, the purpose of the present study was to investigate the role of these enzymes in the pathogenesis of these bullous dermatoses. Studies examining MMPs (matrix metalloproteinases) and the ADAM (a disintegrin and metalloproteinase) family of proteases in pemphigus and pemphigoid were selected from articles published in the repository of the National Library of Medicine (MEDLINE/PubMed) and bioRxiv. Multiple phases of screening were conducted, and relevant data were extracted and tabulated, with 29 articles included in the final qualitative analysis. The majority of the literature investigated the role of specific components of the MMP family primarily in bullous pemphigoid (BP) whereas studies that focused on pemphigus were rarer. The most commonly studied metalloproteinase was MMP-9 followed by MMP-2; other MMPs included MMP-1, MMP-3, MMP-8, MMP-12 and MMP-13. Molecules related to MMPs were also included, namely, ADAM5, 8, 10, 15, 17, together with TIMP-1 and TIMP-3. The results demonstrated that ADAM10 and MMP-9 activity is necessary for blister formation in experimental models of pemphigus vulgaris (PV) and BP, respectively. The data linking MMPs to the pathogenesis of experimental BP were relatively strong but the evidence for involvement of metalloproteinases in PV was more tentative. These molecules represent potential candidates for the development of mechanism-based treatments of these blistering diseases.

## 1. Introduction

Pemphigus and pemphigoid are members of a heterogeneous group of chronic, potentially fatal autoimmune diseases associated with blistering of the skin and mucosae. Once considered part of the same disease spectrum, these bullous dermatoses were first classified as distinct entities by Lever in 1953 [1]. Histologically, pemphigus presents as an intraepithelial split (acantholysis) whereas in pemphigoid, the blisters develop at the dermo-epidermal junction resulting in a subepithelial split.

The two major pemphigus variants, pemphigus vulgaris and pemphigus foliaceus, account for 90–95% of diagnoses [2,3]. Pemphigus diseases are characterized by flaccid blisters and erosions and are treated with high dose corticosteroids and immunosuppressants. By contrast, pemphigoid presents with tense blisters and erosions, a negative Nikolsky sign and, commonly, is controlled using topical steroids although in more severe cases, systemic steroids are an option [2]. 

The development of molecular techniques has facilitated the further classification of these blistering diseases. The pemphigoid group is now known to include at least eight disorders for which the molecular target antigens have been identified [4], although controversy exists regarding the pathogenic mechanisms of blistering [5]. In pemphigus, distinct autoantibody profiles and, in particular, the presence/absence and type of antibodies to cadherins determines the acantholytic signaling pathways [6,7] and possibly the clinical phenotype [8,9]. Despite advances in understanding the pathophysiology of these disorders, however, the mainstay of treatment is the use of corticosteroids and immune suppressants and, significantly, no mechanism-based treatments have been successfully translated to patient care. 

Pemphigus and pemphigoid result from a deficit in cellular adhesion. In pemphigus, cadherin-mediated cell-to-cell adhesion is disrupted due to antibody-mediated signaling pathways and this, in turn, leads to cell-cell detachment (acantholysis) and blister formation [10]. In pemphigoid, both autoantibody and cell-mediated responses induce a disruption of integrin-mediated adhesion between basal keratinocytes and the extracellular matrix (ECM) of the basement membrane [5]. One of the possible mechanisms for the weakening of cell adhesion in these circumstances is that cadherins and/or integrins are digested by proteolytic cleavage [11,12].

Metalloproteinases are proteolytic enzymes with a relatively large spectrum of activity that includes adhesion molecules [13]. Of these, matrix metalloproteinases (MMPs, also known as matrixins) and a disintegrin and metalloproteinase (ADAM) family of endopeptidases, are capable of degrading all kinds of extracellular matrix proteins and extracellular portions of transmembrane proteins, respectively [14]. Metalloproteinases are secreted by both keratinocytes and infiltrating immune cells and modulate a number of physiological and pathological processes in the skin and mucous membranes [15,16]. It is possible, therefore, that these enzymes play a fundamental role in mediating the disruption of intercellular and/or cell-ECM adhesion.

The purpose of the present scoping review was to gather evidence for the involvement of MMPs and ADAMs in the pathophysiology of pemphigus and pemphigoid.

## 2. Methods

### 2.1. Search Strategy

The review was designed to analyze the role of metalloproteinases in the autoimmune blistering diseases pemphigus and pemphigoid. Evidence was gathered for a variety of experimental settings (in vitro, in vivo, and human studies) from peer-reviewed articles published in MEDLINE/PubMed, as well as from pre-prints available in bioRxiv. The results were reported according to the methodology outlined by the most recent Preferred Reporting Items for Systematic Reviews and Meta-analyses extension for Scoping Reviews (PRISMA-ScR) guidelines [17].

The search was conducted in August 2021 and the following search string was applied to the databases:

(metalloproteinase OR metalloprotease OR gelatinase OR disintegrin OR ADAM) and (Pemphigus OR Pemphigoid).

### 2.2. Eligibility Criteria

As this review aimed to determine the role of metalloproteinases in autoimmune blistering diseases, articles were included if they involved either disease specimens or disease models and were related to metalloproteinases. Studies were excluded if they were not in English, were review articles, reported on non-autoimmune blistering diseases or did not assess MMPs or ADAMs. There was no time restriction. Only autoimmune forms of pemphigus (i.e., vulgaris, vegetans, foliaceus, erythematosus) were included, whereas non-immune-mediated forms such as Hailey–Hailey disease (benign chronic familial pemphigus) were excluded.

### 2.3. Data Selection, Collection and Synthesis

After applying the exclusion criteria, a title and abstract screening was conducted to determine if an article was relevant. Articles were excluded if they did not mention pemphigus, pemphigoid, or metalloproteinases. Adhering to the Cochrane guidelines, a lenient policy was adopted during the selection process where there was any uncertainty. In the final phase, full-text screening of each article was performed by two independent reviewers. Data were extracted from the selected articles and tabulated. The main outcomes were evaluated on the basis of (1) the expression of metalloproteinases in pemphigus and pemphigoid, and (2) whether these metalloproteinases were essential for disease development. The pathogenicity criterion was satisfied if the study met the following: (i) serum, IgG, or disease-specific monoclonal antibodies induced pemphigus or pemphigoid-like phenotype in vivo or cell-cell or cell-ECM detachment in vitro; or study in humans; and (ii) a specific pharmacological block, knock-out, knock-down, silencing or inactivation, knock-in or activation, of metalloproteinases prevented or significantly reduced disease phenotype in vivo, cell detachment in vitro, or ameliorated blistering/clinical manifestations in pemphigus or pemphigoid patients.

## 3. Results

### 3.1. Overview of the Search Process

**Figure 1:**
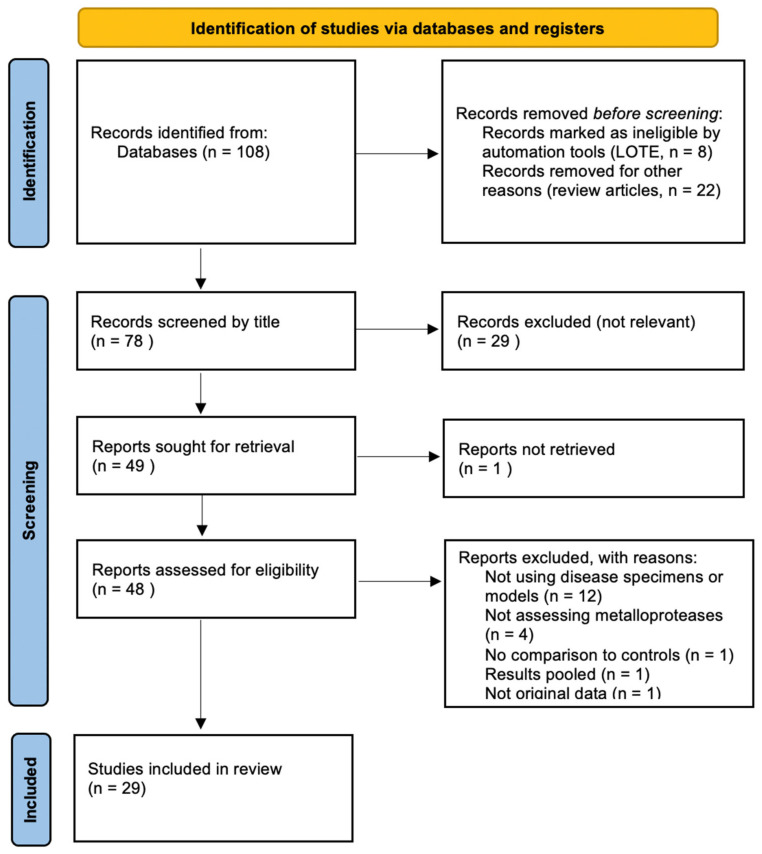
Flowchart of data selection process in accordance with PRISMA-ScR guidelines.

Following the implementation of the search strategy, 108 titles were identified on databases. Of these, 29 articles [18,19,20,21,22,23,24,25,26,27,28,29,30,31,32,33,34,35,36,37,38,39,40,41,42,43,44,45,46] met the inclusion criteria and were included in the qualitative synthesis (Figure 1).

The majority of the selected studies focused on pemphigoid (n = 27) whereas only 5 studies were related to pemphigus (Table 1). The most common pemphigoid subtype was bullous pemphigoid (BP, n = 24), followed by ocular mucous membrane (cicatricial) pemphigoid (OCP, n = 4). Pemphigus vulgaris (PV) was exclusively examined in the pemphigus category. In seven studies, two or more blistering diseases were assessed for the expression of metalloproteinases, and diseases also included the autoimmune skin disorders dermatitis herpetiformis (DH, n = 4) and epidermolysis bullosa acquisita (EBA, n = 2).

The expression of MMPs and ADAMs was examined by immunohistochemistry, western blotting, protein arrays, ELISA, DNA microarrays, PCR, and in situ hybridization; MMP2/9 activity was investigated using gelatin zymography and colorimetric MMP assays (Supplementary Table S1). The most commonly studied metalloproteinase was MMP-9 (n = 23) and in six articles, MMP-2 was also investigated. The expression of other MMPs (MMP-1, MMP-3, MMP-8, MMP-12, and MMP-13), ADAM family members (ADAM10 and ADAM17) and TIMPs (TIMP-1) were also reported.

### 3.2. Summary of Findings

The majority of studies (n = 18) used human samples and assessed metalloproteinase expression in patients’ skin and blister fluid. In vitro and in vivo models were used in 15 and six studies, respectively. In vitro experiments were often carried out in association with either human or animal studies (n = 8). In general, the expression of MMPs was dysregulated in lesional skin of patients with autoimmune blistering diseases. MMP-9 activity was found to be pathogenic in animal models of BP [38,42]. Specifically, mice deficient in functional MMP-9 failed to develop skin blisters in response to anti-mBP180 IgG [42]. In PV, inhibition of ADAM10 prevented blister formation in mice injected with PV IgG that did not contain anti-Dsc antibodies [21].

For a detailed description of individual studies, see Table 1 and Supplementary Table S1.

**Table 1:** Summary of studies assessing metalloproteinases in pemphigus and pemphigoid.

Ref.	First Author, Year	Type	Disease	Target
[18]	Woodward et al., 2020	Human	OCP	MMP9
[19]	Le Jan et al., 2019	In vitro	BP	MMP9
[20]	Riani et al., 2019	In vitro, human	BP	MMP9
[21]	Ivars et al., 2020	In vivo	PV	ADAM10
[22]	de Graauw et al., 2018	In vitro	BP	MMP2/9
[23]	Shen et al., 2018	Human	BP	ADAM10
[24]	Riani et al., 2017	In vitro	BP	MMP9
[25]	Fujimura et al., 2017	Human	PV, BP	MMP12
[26]	Zebrowska et al., 2014	Human	BP, DH	MMP9
[27]	Massie et al., 2015	In vitro	OCP	MMP2/9
[28]	Le Jan et al., 2014	In vitro, human	BP	MMP9
[29]	Arafat et al., 2014	Human	BP	MMP8, MMP9, TIMP1
[30]	Zebrowska et al., 2012	Human	BP, DH	ADAM17
[31]	Oswald et al., 2012	In vitro	BP	MMP9
[32]	Lin et al., 2011	In vitro, in vivo	BP	MMP9
[33]	Chan et al., 2011	Human	OCP	MMP9
[34]	Saw et al., 2011	In vitro	OCP	MMP3/8/13
[35]	Zebrowska et al., 2009	Human	BP, DH	ADAM8/15/17
[36]	Cirillo et al., 2007	In vitro, in vivo	PV	TIMP3, ADAM5, MMP9
[37]	Niimi et al., 2006	Human	BP	MMP2/9/13
[38]	Liu et al., 2005	In vitro, in vivo	BP	MMP3/9
[39]	Shimanovich et al., 2004	In vitro	BP, EBA	MMP9
[40]	Verraes et al., 2001	In vitro, human	BP	MMP2/9, TIMP1
[41]	Liu et al., 2000	In vitro, in vivo	BP	MMP9
[42]	Liu et al., 1998	In vivo	BP	MMP9
[43]	Saarialho-Kere et al., 1995	Human	EB, PV, BP	MMP1
[44]	Ståhle-Bäckdahl et al., 1994	In vitro, human	BP	MMP9
[45]	Oikarinen et al., 1993	Human	BP	MMP2/9
[46]	Oikarinen et al., 1983	Human	BP, DH, PV	

BP, bullous pemphigoid; OCP, ocular cicatricial pemphigoid; PV, pemphigus vulgaris; DH, dermatitis herpetiformis; EBA, epidermolysis bullosa acquisita.

## 4. Discussion

The purpose of this scoping review was to assess the role of metalloproteinases in the pathogenesis of pemphigus and pemphigoid. Despite the relatively limited evidence that is available, a pathogenic role for MMPs and ADAMs in mouse models could successfully be established for both autoimmune blistering diseases.

### 4.1. MMP-9 and Pemphigoid

An array of descriptive research linking metalloproteinases, particularly MMP-9, to pemphigoid has already been published but there remains a paucity of mechanistic studies. Early work reported 72 and 92 kd collagenases, now known as MMP-2 and MMP-9, in skin blisters of pemphigoid patients [46]. MMP-2 was thought to be produced predominantly by fibroblasts whereas MMP-9 was localized to the epidermis and endothelial cells [45]; later, MMP-9 was also found to be secreted by eosinophils at the site of blister formation [40]. More recently, other sources of MMP-9 in BP blisters have been identified including T cells and monocyte-derived macrophages [20]. It is now well documented that MMP-2 is constitutively expressed in several tissues whereas MMP-9 expression appears to be regulated by a number of cell type-specific signaling pathways, for example during inflammation and in response to a changing redox state [47]. This is consistent with the fact that MMP-9 is highly expressed in inflammatory cells, including macrophages, lymphocytes, neutrophils, and eosinophils [48], which are found in the inflammatory infiltrate of BP blisters.

In a landmark study in 1998, a genetically engineered mouse featuring targeted disruption of the *gelatinase B* (*GB*; *MMP*-*9*; 92 kD gelatinase) gene was used to explore the immunopathogenesis of pemphigoid [42]. *GB*-deficient mice were resistant to the blistering effect of intracutaneous anti-mBP180 antibodies but when the mice were reconstituted with normal neutrophils, blister formation occurred, thus implicating neutrophil-derived GB/MMP-9 in the pathogenesis of experimental pemphigoid. A subsequent study from the same group used *GB* −/− and *neutrophil elastase* (*NE*) −/− mice to show that *GB* acts upstream to regulate *NE* activity by inactivating alpha1-proteinase inhibitor and that only *NE*, but not *GB*/*MMP*-*9*, degrades BP180 to produce a dermal-epidermal separation in vivo [41]. This pathogenic pathway was further categorized by the demonstration that the plasminogen (Plg) cascade was also involved in Pemphigoid blister formation. In particular, *Plg*-deficient mice reconstituted locally with the active form of MMP-9, but not the proenzyme form of MMP-9, develop pemphigoid, thus showing that the Plg/plasmin system is epistatic to MMP-9 activation and subsequent dermal-epidermal separation [38].

Mechanistically, MMP-9 has been shown to cleave the extracellular domain of the 180 kd bullous pemphigoid autoantigen in vitro [44], but not in vivo [41], thus questioning its direct role in causing the dermo-epidermal split. It is worth noting that MMP-9 can cleave BP180 into small tripeptides, which significantly enhance neutrophil chemotaxis and NE release in vivo [28]. Therefore, it is likely that the effects of MMP-9 overexpression in BP are more prominent upstream of the degradation of BP180 and that MMP-9 acts by amplifying the inflammatory cascade and activating proteolytic enzymes, whereas NE is the final executioner of BP180 cleavage [49].

While considerable progress has been made regarding the role of MMPs in the pathogenesis of experimental pemphigoid, no mechanism-based treatments for the disease have been developed to date. This may partially be due to the difference between experimental models and human disease. MMP-9, for example, is essential for blister formation induced by anti-BP180 antibodies in mice, but pemphigoid patients develop autoantibodies against other junctional molecules including BP230, laminin 311, type VII collagen and α6β4 integrin [4,5]. While MMP-9 can target ECM proteins such as type VII collagen and β4 integrin in vitro [50], the capacity of MM9 to induce a dermo-epidermal split in vivo remains to be established.

Interestingly, only the proenzymes of MMP-2/9 could be found in lesional skin and in blister fluid of pemphigoid patients [40] which contrasts to the situation in experimental mice where both the pro- and active forms of MMP-9 were detected in lesional and non-lesional skin samples [32]. This raises the question as to whether the pathophysiological events underlying MMP-9 activation in mouse models also takes place in BP in humans.

Taken together with the lack of clinical translation of MMP inhibitors for the treatment of BP patients, these data suggest that the pathogenic role of MMP-9 in human BP may be more complex than previously anticipated in animal models.

### 4.2. Metalloproteinases in Pemphigus

Despite the well-known ability of metalloproteinases to cleave the extracellular domain of desmogleins (Dsgs) and other cell adhesion molecules [51,52,53], data relating to their involvement in the pathogenesis of PV are scanty. An increase in the expression of ADAM5 and MMP-9, together with the downregulation of TIMP3, occurs in the skin of mice injected with PV sera [36]. Further, MMP-12 has been detected in the upper layer of epidermis and superficial dermis in PV lesions [25]. Interestingly, PV IgG triggers a significant increase in ADAM10 activity in a Src-dependent manner; inhibition of ADAM10 prevents histological acantholysis and skin blistering in mice injected with IgG from PV patients with a typical autoantibody profile, but not with PV IgG containing antibodies to desmocollins [21]. The authors propose that anti-Dsg IgG-dependent ADAM10 activity leads to EGFR activation and acantholysis but it is also known that ADAM10 targets epithelial cell adhesion molecules by direct cleavage [54]. Overall, these findings indicate that anti-Dsg1/3 antibodies trigger a signaling pathway culminating in pathogenic ADAM10 activation, whereas non-Dsg antibodies in PV induce blistering in an ADAM10-independent manner.

### 4.3. Limitations and Future Directions

This study has limitations in terms of internal and external validity. The use of only one search engine (PubMed) for peer-reviewed articles together with one pre-print server (bioRxiv) may have failed to identify relevant studies from other databases. Furthermore, our search string focused on a relatively new nomenclature. MMP-1 and MMP-8, for example, were once known as interstitial collagenase and neutrophil collagenase, respectively, and this may have resulted in the exclusion of older studies. Finally, some articles may have been overlooked due to the strict exclusion criteria adopted and the search string used.

The differences between passive transfer mouse models and human disease may hinder the potential translational applications of our results. For example, despite the important role of MMPs in many human diseases, none of the broad-range synthetic MMP inhibitors that have been designed have successfully passed clinical trials [55], partly due to broad-spectrum inhibition of MMPs resulting in severe side effects. Novel classes of selective inhibitors against gelatinases, collagenases, membrane-type and elastase subgroups are being developed [56] and will likely pave the path to new therapeutic strategies.

## 5. Conclusions

Despite the relatively limited number of studies investigating the role of MMPs in autoimmune blistering diseases, inactivation of ADAM10 and MMP-9 were shown to prevent blister formation in experimental models of pemphigus and pemphigoid, respectively. These molecules and their related pathways represent potential candidates for clinical translation and for developing mechanism-based treatments of these conditions.

## Data Availability

Supplementary results are available with the article. All data items not included in the manuscript are available upon reasonable request to the corresponding author.

## Supplementary Materials

The following are available online at https://www.mdpi.com/article/10.3390/biom11101506/s1, Table S1: Detailed list of included studies.

## References

[1] Lever W.F. (1953). Pemphigus. Medicine.

[2] Schmidt E., Kasperkiewicz M., Joly P. (2019). Pemphigus. Lancet.

[3] Bystryn J.C., Rudolph J.L. (2005). Pemphigus. Lancet.

[4] Schmidt E., Zillikens D. (2013). Pemphigoid diseases. Lancet.

[5] Genovese G., Di Zenzo G., Cozzani E., Berti E., Cugno M., Marzano A.V. (2019). New Insights Into the Pathogenesis of Bullous Pemphigoid: 2019 Update. Front Immunol..

[6] Walter E., Vielmuth F., Rotkopf L., Sárdy M., Horváth O.N., Goebeler M., Schmidt E., Eming R., Hertl M., Spindler V. (2017). Different signaling patterns contribute to loss of keratinocyte cohesion dependent on autoantibody profile in pemphigus. Sci. Rep..

[7] Chernyavsky A., Patel K.G., Grando S.A. (2020). Mechanisms of synergy of autoantibodies to M3 muscarinic acetylcholine receptor and secretory pathway Ca2+/Mn2+-ATPase isoform 1 in patients with non-desmoglein pemphigus vulgaris. Int. Immunopharmacol..

[8] Oktem A., Hayran Y., Uysal P.İ., Atılan A.U., Yalçın B. (2018). Evaluation of the Importance of Immunological Profile for Pemphigus Vulgaris in the Light of Necessity to Modify Compensation Theory. Acta Dermatovenerol. Croat..

[9] Gualtieri B., Marzano A.V., Grando S. (2020). Atypical pemphigus: Autoimmunity against desmocollins and other non-desmoglein autoantigens. Ital. J. Dermatol. Venereol..

[10] Kaur B., Kerbrat J., Kho J., Kaler M., Kanatsios S., Cirillo N. (2021). Mechanism-based therapeutic targets of pemphigus vulgaris: A scoping review of pathogenic intracellular pathways. Exp. Dermatol..

[11] Gornowicz-Porowska J., Bowszyc-Dmochowska M., Dmochowski M. (2011). Autoimmunity-driven enzymatic remodeling of the dermal–epidermal junction in bullous pemphigoid and dermatitis herpetiformis. Autoimmunity.

[12] Cirillo N., Dell’Ermo A., Gombos F., Lanza A. (2008). The specific proteolysis hypothesis of pemphigus: Does the song remain the same?. Med. Hypotheses.

[13] Rawlings N.D., Barrett A.J. (1995). [13] Evolutionary families of metallopeptidases. Meth. Enzymol..

[14] Amar S., Minond D., Fields G.B. (2017). Clinical Implications of Compounds Designed to Inhibit ECM-Modifying Metalloproteinases. Proteomics.

[15] Krishnaswamy V.R., Mintz D., Sagi I. (2017). Matrix metalloproteinases: The sculptors of chronic cutaneous wounds. Biochim. Biophys. Acta (BBA) Bioenerg..

[16] Riihilä P., Nissinen L., Kähäri V. (2020). Matrix metalloproteinases in keratinocyte carcinomas. Exp. Dermatol..

[17] Page M.J., McKenzie J.E., Bossuyt P.M., Boutron I., Hoffmann T.C., Mulrow C.D., Shamseer L., Tetzlaff J.M., Akl E.A., Brennan S.E. (2021). The PRISMA 2020 statement: An updated guideline for reporting systematic reviews. BMJ.

[18] Woodward A.M., Di Zazzo A., Bonini S., Argüeso P. (2020). Endoplasmic reticulum stress promotes inflammation-mediated proteolytic activity at the ocular surface. Sci. Rep..

[19] Le Jan S., Muller C., Plée J., Durlach A., Bernard P., Antonicelli F. (2019). IL-23/IL-17 Axis Activates IL-1β-Associated Inflammasome in Macrophages and Generates an Auto-Inflammatory Response in a Subgroup of Patients With Bullous Pemphigoid. Front Immunol..

[20] Riani M., Muller C., Bour C., Bernard P., Antonicelli F., Le Jan S. (2019). Blister Fluid Induces MMP-9-Associated M2-Type Macrophages in Bullous Pemphigoid. Front. Immunol..

[21] Ivars M., España A., Alzuguren P., Pelacho B., Lasarte J., López-Zabalza M. (2019). The involvement of ADAM 10 in acantholysis in mucocutaneous pemphigus vulgaris depends on the autoantibody profile of each patient. Br. J. Dermatol..

[22] De Graauw E., Sitaru C., Horn M.P., Borradori L., Yousefi S., Simon D., Simon H.U. (2018). Monocytes enhance neutrophil-induced blister formation in an ex vivo model of bullous pemphigoid. Allergy.

[23] Shen S., Ke Y., Dang E., Fang H., Chang Y., Zhang J., Zhu Z., Shao S., Qiao P., Zhang T. (2018). Semaphorin 4D from CD15+ Granulocytes via ADAM10-Induced Cleavage Contributes to Antibody Production in Bullous Pemphigoid. J. Investig. Dermatol..

[24] Riani M., Le Jan S., Plée J., Durlach A., Le Naour R., Haegeman G., Bernard P., Antonicelli F. (2017). Bullous pemphigoid outcome is associated with CXCL10-induced matrix metalloproteinase 9 secretion from monocytes and neutrophils but not lymphocytes. J. Allergy Clin. Immunol..

[25] Fujimura T., Kakizaki A., Furudate S., Aiba S. (2017). A possible interaction between periostin and CD163(+) skin-resident macro-phages in pemphigus vulgaris and bullous pemphigoid. Exp. Dermatol..

[26] Żebrowska A., Wagrowska-Danilewicz M., Danilewicz M., Stasikowska-Kanicka O., Kulczycka-Siennicka L., Woźniacka A., Waszczykowska E. (2014). Mediators of Mast Cells in Bullous Pemphigoid and Dermatitis Herpetiformis. Mediat. Inflamm..

[27] Massie I., Dale S.B., Daniels J.T. (2015). Limbal Fibroblasts Maintain Normal Phenotype in 3D RAFT Tissue Equivalents Suggesting Po-tential for Safe Clinical Use in Treatment of Ocular Surface Failure. Tissue Eng. Part C Methods.

[28] Le Jan S., Plée J., Vallerand D., Dupont A., Delanez E., Durlach A., Jackson P.L., Blalock J.E., Bernard P., Antonicelli F. (2014). Innate immune cell-produced IL-17 sustains inflammation in bullous pemphigoid. J. Investig. Dermatol..

[29] Arafat S.N., Suelves A.M., Spurr-Michaud S., Chodosh J., Foster C.S., Dohlman C.H., Gipson I.K. (2013). Neutrophil Collagenase, Gelatinase, and Myeloperoxidase in Tears of Patients with Stevens-Johnson Syndrome and Ocular Cicatricial Pemphigoid. Ophthalmology.

[30] Zebrowska A., Wagrowska-Danilewicz M., Danilewicz M., Sokolowska M., Stasikowska-Kawecka O., Erkiert-Polguj A., Cynkier A., Pawliczak R., Sysa-Jedrzejowska A., Waszczykowska E. (2012). Does Adam17 cause the destruction of anchoring fibers via shedding tumor necrosis factor α in bullous pemphigoid and dermatitis herpetiformis?. J. Cutan. Med. Surg..

[31] Oswald E., Sesarman A., Franzke C.W., Wölfle U., Bruckner-Tuderman L., Jakob T., Martin S.F., Sitaru C. (2012). The flavonoid luteolin inhibits Fcγ-dependent respiratory burst in granulocytes, but not skin blistering in a new model of pemphigoid in adult mice. PLoS ONE.

[32] Lin L., Bankaitis E., Heimbach L., Li N., Abrink M., Pejler G., An L., Diaz L., Werb Z., Liu Z. (2011). Dual Targets for Mouse Mast Cell Protease-4 in Mediating Tissue Damage in Experimental Bullous Pemphigoid. J. Biol. Chem..

[33] Chan M.F., Sack R., Quigley D.A., Sathe S., Vijmasi T., Li S., Holsclaw D., Strauss E.C., McNamara N.A. (2011). Membrane Array Analysis of Tear Proteins in Ocular Cicatricial Pemphigoid. Optom. Vis. Sci..

[34] Saw V.P., Schmidt E., Offiah I., Galatowicz G., Zillikens D., Dart J.K., Calder V.L., Daniels J.T. (2011). Profibrotic Phenotype of Conjunctival Fibroblasts from Mucous Membrane Pemphigoid. Am. J. Pathol..

[35] Żebrowska A., Wagrowska-Danilewicz M., Danilewicz M., Wodz K., Sokolowska M., Erkiert-Polguj A., Sysa-Jedrzejowska A., Waszczykowska E., Pawliczak R. (2009). Expression of selected ADAMs in bullous pemphigoid and dermatitis herpetiformis. J. Dermatol. Sci..

[36] Cirillo N., Lanza M., Rossiello L., Gombos F., Lanza A. (2007). Defining the involvement of proteinases in pemphigus vulgaris: Evi-dence of matrix metalloproteinase-9 overexpression in experimental models of disease. J. Cell. Physiol..

[37] Niimi Y., Pawankar R., Kawana S. (2006). Increased Expression of Matrix Metalloproteinase-2, Matrix Metalloproteinase-9 and Matrix Metalloproteinase-13 in Lesional Skin of Bullous Pemphigoid. Int. Arch. Allergy Immunol..

[38] Liu Z., Li N., Diaz L.A., Shipley M., Senior R.M., Werb Z. (2005). Synergy between a plasminogen cascade and MMP-9 in autoimmune disease. J. Clin. Investig..

[39] Shimanovich I., Mihai S., Oostingh G.J., Ilenchuk T.T., Bröcker E.-B., Opdenakker G., Zillikens D., Sitaru C. (2004). Granulocyte-derived elastase and gelatinase B are required for dermal–epidermal separation induced by autoantibodies from patients with epidermolysis bullosa acquisita and bullous pemphigoid. J. Pathol..

[40] Verraes S., Hornebeck W., Bernard P., Polette M., Borradori L. (2001). Respective contribution of neutrophil elastase and matrix met-alloproteinase 9 in the degradation of BP180 (type XVII collagen) in human bullous pemphigoid. J. Investig. Dermatol..

[41] Liu Z., Zhou X., Shapiro S.D., Shipley J., Twining S.S., Diaz L.A., Senior R.M., Werb Z. (2000). The Serpin α1-Proteinase Inhibitor Is a Critical Substrate for Gelatinase B/MMP-9 In Vivo. Cell.

[42] Liu Z., Shipley J.M., Vu T.H., Zhou X., Diaz L.A., Werb Z., Senior R.M. (1998). Gelatinase B–deficient Mice Are Resistant to Experimental Bullous Pemphigoid. J. Exp. Med..

[43] Saarialho-Kere U.K., Vaalamo M., Airola K., Niemi K.-M., Oikarinen A.I., Parks W.C. (1995). Interstitial Collagenase Is Expressed by Keratinocytes That Are Actively Involved in Reepithelialization in Blistering Skin Diseases. J. Investig. Dermatol..

[44] Ståhle-Bäckdahl M., Inoue M., Guidice G.J., Parks W.C. (1994). 92-kD gelatinase is produced by eosinophils at the site of blister formation in bullous pemphigoid and cleaves the extracellular domain of recombinant 180-kD bullous pemphigoid autoantigen. J. Clin. Investig..

[45] Oikarinen A., Kylmäniemi M., Autio-Harmainen H., Autio P., Salo T. (1993). Demonstration of 72-kDa and 92-kDa Forms of Type IV Collagenase in Human Skin: Variable Expression in Various Blistering Diseases, Induction During Re-Epithelialization, and Decrease by Topical Glucocorticoids. J. Investig. Dermatol..

[46] Olkarinen A.I., Zone J.J., Ahmed A.R., Kiistala U., Uitto J., Olkarinen J.J.Z.A.I. (1983). Demonstration of Collagenase and Elastase Activities in the Blister Fluids from Bullous Skin Diseases. Comparison Between Dermatitis Herpetiformis and Bullous Pemphigoid. J. Investig. Dermatol..

[47] Cancemi P., Aiello A., Accardi G., Caldarella R., Candore G., Caruso C., Ciaccio M., Cristaldi L., Di Gaudio F., Siino V. (2020). The Role of Matrix Metalloproteinases (MMP-2 and MMP-9) in Ageing and Longevity: Focus on Sicilian Long-Living Individuals (LLIs). Mediat. Inflamm..

[48] Visse R., Nagase H. (2003). Matrix metalloproteinases and tissue inhibitors of metalloproteinases: Structure, function, and biochem-istry. Circ. Res..

[49] Lin L., Betsuyaku T., Heimbach L., Li N., Rubenstein D., Shapiro S.D., Liu Z. (2012). Neutrophil elastase cleaves the murine hemidesmosomal protein BP180/type XVII collagen and generates degradation products that modulate experimental bullous pemphigoid. Matrix Biol..

[50] Pal-Ghosh S., Blanco T., Tadvalkar G., Pajoohesh-Ganji A., Parthasarathy A., Zieske J., Stepp M.A. (2011). MMP9 cleavage of the β4 integrin ectodomain leads to recurrent epithelial erosions in mice. J. Cell Sci..

[51] Klessner J.L., Desai B.V., Amargo E.V., Getsios S., Green K.J. (2009). EGFR and ADAMs Cooperate to Regulate Shedding and Endocytic Trafficking of the Desmosomal Cadherin Desmoglein 2. Mol. Biol. Cell.

[52] Cirillo N., Femiano F., Gombos F., Lanza A. (2006). Metalloproteinase 9 is the outer executioner of desmoglein 3 in apoptotic keratinocytes. Oral Dis..

[53] Weiske J., Schöneberg T., Schröder W., Hatzfeld M., Tauber R., Huber O. (2001). The Fate of Desmosomal Proteins in Apoptotic Cells. J. Biol. Chem..

[54] Bech-Serra J.J., Santiago-Josefat B., Esselens C., Saftig P., Baselga J., Arribas J., Canals F. (2006). Proteomic Identification of Desmoglein 2 and Activated Leukocyte Cell Adhesion Molecule as Substrates of ADAM17 and ADAM10 by Difference Gel Electrophoresis. Mol. Cell. Biol..

[55] Levin M., Udi Y., Solomonov I., Sagi I. (2017). Next generation matrix metalloproteinase inhibitors—Novel strategies bring new pro-spects. Biochim. Biophys. Acta Mol. Cell. Res..

[56] Lenci E., Cosottini L., Trabocchi A. (2021). Novel matrix metalloproteinase inhibitors: An updated patent review (2014–2020). Expert Opin. Ther. Patents.

